# Chemical disruption of placental thyroid hormone signalling: a systematic review that highlights sex-specific effects

**DOI:** 10.1007/s00204-025-04203-z

**Published:** 2025-09-25

**Authors:** Julia Swan, D. Zhurenko, K. M. Huttunen, J. Rysä

**Affiliations:** School of Pharmacy, Faculty of Health Sciences, University of Eastern Finland, Yliopistonrinne 3, P.O.Box 1627, 70211 Kuopio, Finland

**Keywords:** Placenta, Thyroid hormone, Chemical exposure, Sex differences

## Abstract

Thyroid hormones are crucial for growth, brain development, metabolism, and organ maturation in developing foetuses. Until 12–14 weeks of gestation, the foetus depends on maternal thyroid hormones before its own thyroid gland begins functioning. Environmental chemical and medication exposure during pregnancy may affect the thyroid hormone supply to the foetus by interfering with placental transport carriers and metabolism. This systematic review evaluated chemical effects on thyroid hormone passage from maternal to foetal circulation, modulated by transporters and enzymes. A search of PubMed, Scopus, and Web of Science identified 24 relevant studies published between 1900 and 2024, including 4 epidemiological studies, 8 in vivo animal studies, and 15 in vitro studies. The review found evidence that persistent organic pollutants, flame retardants, endocrine disrupting chemicals, pharmaceuticals, and other substances can disrupt placental thyroid hormone signalling through various mechanisms. These include alterations in transporter expression and enzyme activity in the placenta. Several studies observed sex-specific effects, with male and female foetuses showing different responses to chemical exposure. In some cases, sex differences were in the degree of change, while in others, the same chemical had opposite effects based on foetal sex. However, many studies used choriocarcinoma cell lines, which may not fully replicate human placental processes. This review highlights the need for further research to elucidate chemical exposure's impact on foetal thyroid hormone status and the role of foetal sex using human physiologically relevant models.

**Supplementary Information:**

The online version contains supplementary material available at 10.1007/s00204-025-04203-z.

## Introduction

Thyroid hormones play a critical role in foetal growth and development, particularly in the developing brain and the nervous system (Moog et al. 2017). During pregnancy, the foetus relies entirely on maternal thyroid hormones in the first trimester and continues to depend on the maternal supply to some degree throughout gestation (Moog et al. 2017). Thyroid hormone homeostasis is essential for normal brain development. Disruptions in thyroid hormone homeostasis during development can lead to significant neurodevelopmental disorders including cognitive impairments, behavioural problems and learning disabilities (O’Shaughnessy and Gilbert 2020). Moreover, the prevalence of conditions related to foetal thyroid disturbances, such as congenital hypothyroidism, has increased over time (Liu et al. 2023; Yao et al. 2023). For example, the overall pooled congenital hypothyroidism prevalence increased from 2.37 per 1000 neonates between 1969 and 1980 to 5.26 per 1000 neonates between 2011 and 2020 (Liu et al. 2023). Previous studies suggest that the upward trend of congenital hypothyroidism incidence may be due to changes in population demographics, the increasing survival rate of preterm infants, and the lowering of thyroid-stimulating hormone (TSH) cutoff values (Harris and Pass 2007; Albert et al. 2012; Olivieri et al. 2015). However, these changes do not fully explain this increase, highlighting the need for further research to fully understand this trend and its implications for diagnosis and treatment.

Under normal circumstances, thyroxine (T4) and triiodothyronine (T3) reach the placenta, bound to serum albumin, thyroxine-binding globulin (TBG), and transthyretin (TTR). They then enter the trophoblast via membrane transporters which include members of the large neutral amino acid transporter family (LAT1 and LAT2), members of the monocarboxylate transporter family (MCT8 and MCT10), and members of the organic anion transporting polypeptides (OATP1A2, OATP4A1, and OATP-E) and are transported to foetal circulation (Zuñiga et al. 2022).

The level of active thyroid hormone (TH) in the placenta can be modulated by several enzymes. Deiodinase 2 (DIO2) catalyses the conversion of T4 into the highly active form of T3. DIO3 catalyses the inactivation of T4 and T3 by converting them into inactive reverse T3 (rT3) and 3,5′-iiodothyronine (T2) forms, respectively. Deactivation of T3 and T4 limits the passage of these hormones from the maternal to the foetal bloodstream. This process controls the foetus’s exposure to T3 and T4, originating from the mother. In addition, enzyme activity also regulates iodine availability in foetal blood, regulating the foetus’s ability to produce its own thyroid hormones.

However, these enzymes are not the only enzymes that regulate the availability of active thyroid hormones in the foetus. Sulfotransferase (SULT) enzymes play a crucial role in regulating placental thyroid hormone levels primarily through sulfation (Zuñiga et al. 2022). Sulfation renders thyroid hormones reversibly inactive, allowing them to contribute to the reserve pool of thyroid hormones. When needed, sulfation is reversed by foetal arylsulfatases, rendering thyroid hormones active (Zuñiga et al. 2022). Abnormal SULT activity could lead to imbalances in the conversion of active to inactive thyroid hormones, which could affect the availability of active thyroid hormones for foetal development.

Several environmental chemicals such as persistent organic pollutants (POPs) can potentially disrupt the thyroid system (Gilbert et al. 2020). These substances have been detected in both maternal and foetal tissues, suggesting widespread exposure during foetal development (Leonetti et al. 2016; Kim et al. 2019). These chemicals can interfere with the synthesis and function of placental TH carriers and metabolising enzymes, leading to disrupted thyroid hormone signalling in the developing foetus.

In this systematic literature review, we investigated the effects of chemicals on the passage of thyroid hormones from maternal to foetal circulation, modulated by plasma membrane transporters, enzymes, and carrier proteins. In addition, this systematic literature review pays particular attention to sex-specific effects and mechanisms of disruption, as emerging evidence suggests significant differences in how placentas from male and female foetuses respond to environmental stressors, such as chemical exposure. This may provide some insight into how chemical exposure during foetal development contributes to the rising prevalence of conditions related to foetal thyroid disturbance.

## Materials and methods

### Search strategy

**Fig. 1:**
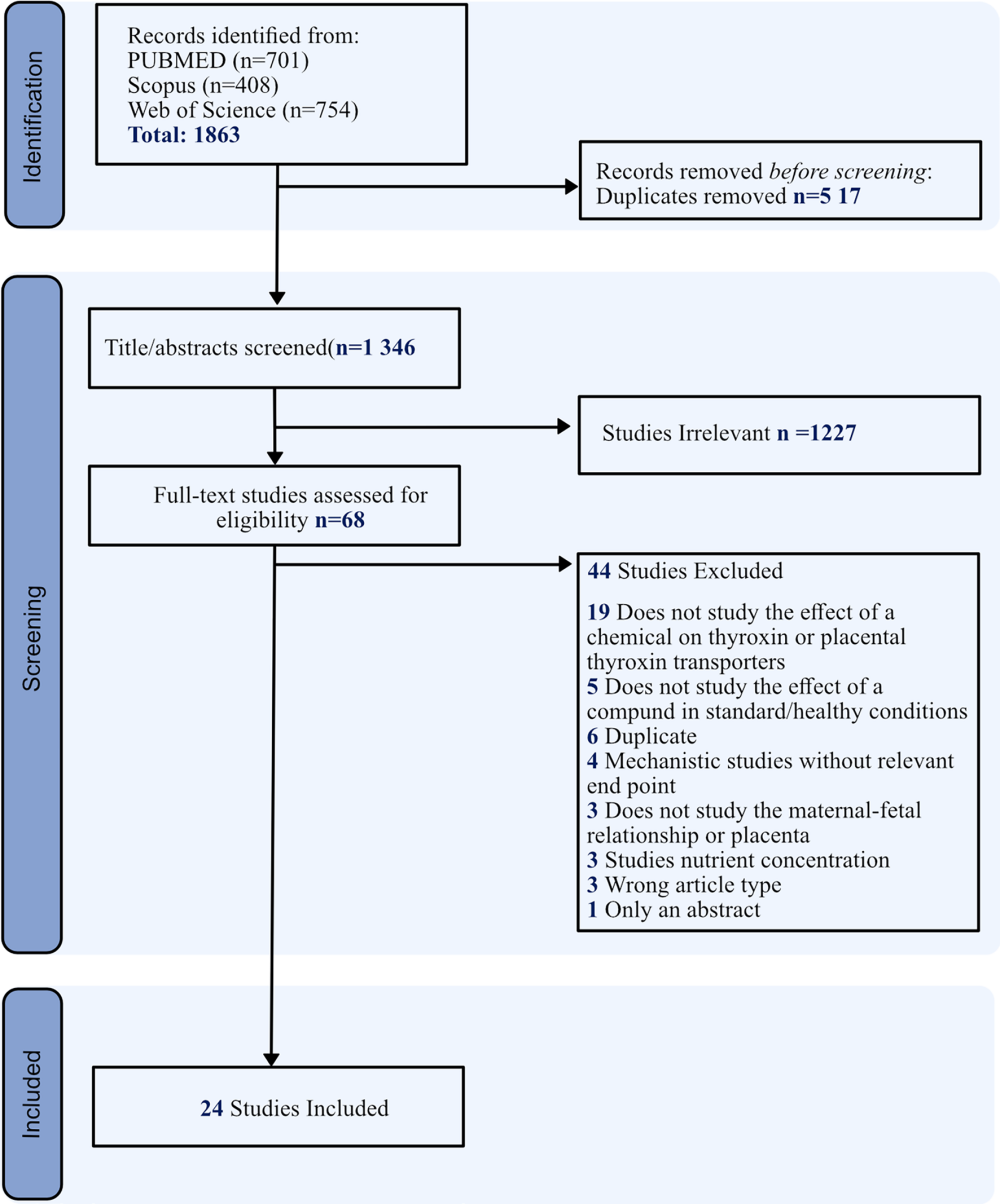
PRISMA flowchart illustrating the qualification process of the included studies for the systematic review

The PRISMA guidelines for reporting were followed. Peer-reviewed publications between 1900 and February 2024 were sourced for this systematic review from PubMed, Scopus, and Web of Science (Fig. 1). The search term strings can be found in Supplemental Table 1.

### Eligibility criteria

After duplicates were removed, the studies were independently screened by two reviewers using a two-phase Covidence Systematic Review platform. First, an initial title and abstract screening was performed, excluding studies that (a) did not describe an original study in mammals or mammalian cells, and (b) did not mention anything related to the thyroid, thyroid hormone, or any of the thyroid hormone placental transporters described by Zuñiga et al. (2022). A study was included in the next phase if there was disagreement between the reviewers or insufficient information for exclusion. A second screening by the two reviewers was performed on the full text according to the predefined inclusion and exclusion criteria (Table 1).

**Table 1:** Predefined inclusion and exclusion criteria

	Inclusion	Exclusion
Population	• Maternal–foetal relationship or placenta is studied	• Does not study maternal–foetal relationship or placenta
Exposure	• Environmental chemicals and pharmaceuticals	• Does not study the effect of a chemical on thyroid hormone or placental thyroid hormone transporters • Studies of nutrient concentration
Context/Comparator	• Studies the effect of a compound in standard conditions/healthy state	• Does not study the effect of a compound in standard conditions e.g. studied under hypoxia or diseased states
Outcome	• Placental thyroid hormone transporters or thyroid hormone transport is studied	• Does not assess thyroid hormone or its transporters
Study Characteristics	• Original article: epidemiological, in vivo, in vitro	• Only an abstract • Review article • Mechanistic studies without relevant endpoint

### Study details and outcome data extraction

Two reviewers independently extracted the data from the 24 publications (Fig. 1), and during this phase, the studies were grouped into three categories: chemicals affecting thyroid hormone transport across the placenta in (a) epidemiological studies, (b) in vivo studies, and (c) in vitro studies (Tables 2, 3, 4, 5). During this stage, two publications (Li et al. 2022; Paul et al. 2023) reported relevant in vivo and in vitro components. The experiments were extracted, and quality assessment was performed separately for the relevant in vitro/in vivo experiments.

**Table 2:** Epidemiological studies

References	Study design	Location	Chemical	Markers	Sample size	Chemical level	Main finding	Infant sex differences observed
Leonetti et al. (2016)	Prospective cohort	USA	Brominated flame retardants	T4, T3, rT3 DIO3 SULT	95	2,4,6-TBP mean 15.4 ng/g lipid BDE-47 mean 5.09 ng/g lipid	2,4,6-TBP were positively associated with T3 concentrations. T3 SULT activity among males was positively associated with BDE exposure. BDE-99 was negatively associated with T3 SULT activity among females	Yes
Kim et al. (2019)	Observational cohort	Korea	POPs	MCT8, TTR, DIO3	106	p,p′-DDE (median, 57.7 ng/g lipid), PCB-153 (9.3 ng/g lipid)	Promoter DNA methylation of DIO3 and MCT8, were associated with prenatal exposure to POPs	Yes
Li et al. (2022)	Case control	China	Cadmium	TR TRβ DIO2	23	Blood concentrations in maternal blood, women with pre-eclampsia mean cadmium concentration 0.6ug/l and no preeclampsia 0.4ug/l	The TRα and TRβ (mRNA) and DIO2 (protein) were decreased in preeclampsia placentas	Not studied
McColl et al. (2023)	Case control	Alaska	Tobacco	LAT1	110	Not measured	Oral tobacco use was associated with significantly increased levels of LAT1 mRNA. Protein expression of LAT1 was not affected by tobacco use	Yes

Abbreviations: *DIO3* Deiodinase 3, *DIO2* Deiodinase 2, *DDE* Dichlorodiphenyldichloroethylene, *LAT1* L-Type Amino Acid Transporter 1, *MCT8* Monocarboxylate transporter 8, *PCB* Polychlorinated biphenyls, *rT3* Reverse Triiodothyronine, *SULT* Sulfotransferase, *BDE* Tetrabromodiphenyl ether, *TR* Thyroid hormone receptor, *T4* Thyroxine, *TBP* Tribromophenol, *T3* Triiodothyronine

**Table 3:** In vivo studies

References	Chemical	Species and Strain	Markers	Sample size	Exposure time	Exposure dose	Placenta/infant finding in exposed group	Infant sex differences observed
Meerts et al. (2002)	4-OH-CB107: PCB metabolite	Rat: Wistar	TSH and TT4	4	GD 10 to 16	14.6 mol (5 mg)/kg/day	Decreased infant TT4, increased infant TSH	Not studied
Shukla et al. (2011)	Alcohol	Rat: Sprague–Dawley	DIO3, TRA1	6	GD 8–21	Liquid diet containing 5% w/v ethanol. Blood alcohol levels 126.5 ± 29.0 mg/dl	Placental protein and mRNA: increased expression of DIO3, decreased expression of TRA1	Yes
Wang et al. (2017)	Fenvalerate	Mouse: ICR	TRA1, TRB1, T4 and TT3	14	GD 0–17; experiment end GD 18	0.2, 2.0, and 20 mg/kg	Placental mRNA: decreased expression TRA1 and TRB1 at all exposure doses; Protein: TRB1 only at 20.0 mg/kg exposure	Yes
Yu et al. (2018)	DEHP	Mouse: ICR	TR	12–15	GD 0 throughout pregnancy	50 or 200 mg/kg/day	Placental mRNA Tra1 and Trb1 decreased Nuclear translocation of placental TRA1 and TRB1 was suppressed	Not studied
Guo et al. (2020)	Dexamthasone	Rat: Specific pathogen-free Wistar	LAT1	11	GD 9 to 20	0.2 or 0.8 mg/kg/day	Placental mRNA and protein expression of LAT1 was increased in female placenta and decreased in the male placenta	Yes
Blanco-Castañeda et al. (2022)	Levetiracetam	Mouse:BALB/c	LAT1, OATP4A1	10	Unclear, appears from age of 21 days until GD 13 and GD 18.Pregnant from 3 months of age	100 mg/kg/day	Placental mRNA: increased expression of Lat1 and Oatp4a1	Not studied
Paul et al. (2023)	Cortisone	Rat: C57BL6/J	Dio2 (gene expression) T3, T4	4- 6	GD 12.5–16.5 or 18.5	50 μg/mL in distilled drinking water	Placental mRNA: Decreased Dio2 expression,	Yes

Abbreviations: *GD* Gestational Day, *DIO3* Deiodinase 2, *DIO2* Deiodinase 2, *DDE* Dichlorodiphenyldichloroethylene, *LAT1* L-Type Amino Acid Transporter 1, *OATP4A1* Organic anion transporting polypeptide 4A1, *rT3* Reverse Triiodothyronine, *SULT* Sulfotransferase, *BDE* Tetrabromodiphenyl ether, *TR* Thyroid hormone receptor, *T4* Thyroxine, *TBP* Tribromophenol, *T3* Triiodothyronine, *TSH* Thyroid stimulating hormone

**Table 4:** In vitro studies

References	Chemical	Cell type	Markers	Sample size	Exposure time	Exposure dose	Placenta measurement/finding
Ramamoorthy et al. (1992)	Forskilin	JAR cells	The LAT system	2 to 5	0,5-16 h	100 μM	Forskolin had no effect on the cellular uptake of leucine which uses the LAT transporters
Gregoraszczuk et al. (2014)	HCBz and PeCBz	Human placental explants	SULT1A	3	6 h, 12, 24 h, 48 h	0, 0.02, 0.2, and 2 ng/ml	HCBz Stimulated SULT1A activity at 24 h for lower doses (0.02 and 0.2 ng/ml) and increased SULT1A protein expression at all doses at 48 and 72 h. PeCBz increased SULT1A activity at 24 h (0.02 ng/ml) and at 48 and 72 h (0.2 ng/ml) and increased protein expression at 0.2 ng/ml at all time points
Rubinchik‐Stern et al. (2015)	Antiepileptic Medications	BeWo cells	LAT 1, OATP1A2	6	2 days and 5 days	1. Valproic acid 42, 86, 166 µg/ml 2. Phenytoin 10 and 20 µg/ml 3. Carbamazepine 6 and 12 µg/ml 4. Lamotrigine 3 and 12 µg/ml, 5. Levetiracetam 1 and 30 µg/ml	1. Valproic acid: mRNA decreased expression LAT1 and OATP1A2. No effect on protein expression 2. Phenytoin: mRNA LAT1 decreased and protein LAT1 increased, 3. Carbamazepine mRNA: OATP1A2 decreased, no effect on the protein leve, 4. Lamotrigine: mRNA increase LAT 1 expression and decrease in OATP1A2 protein expression but no dose–response curve 5. Levetiracetam: mRNA decrease in OATP1A2. Decrease in OATP1A2 and LAT1 protein expression with the OATP1A2 not showing a dose response
Balthasar et al. (2017)	Forskolin	BeWo cells	LAT1 and LAT2	not mentioned	24–−72 h	20 mM	LAT1 and LAT2 expression (protein and mRNA) significantly increased after 48 h of Forskolin treatment
Leonetti et al. (2018)	Brominated Flame Retardants	BeWo cells	SULT1A activity and gene expression of, DIO3, SULT1A1, TRA, and TRB	6	1, 6, 12, or 24 h	Low (0.05 µM/0.001 µM), med (0.5 µM), high (1 µM)	2,4,6-TBP, BDE-99, 3-OH BDE-47, and 6-OH BDE-47 decreased SULT activity. No significant changes in mRNA expression for SULT1A1, TR-α, and TR-β in any treatment groups. DIO3 gene expression was highly variable
Qin et al. (2021)	BDE209	JEG-3 cells	DIO2, DIO3 and changes of extracellular TH levels	3	24 h	0.3 and 1 μM	Decreased expression DIO3 (protein and mRNA), increased DIO2 mRNA expression and increased extracellular T4
Du et al. (2020)	DEHP	HTR-8/SVneo cells and JEG-3 cells	TTR, MCT8 and T3,T4 uptake	3	24 h	0, 4, 40, 100 and 400 µM	Reduced TTR mrNA and Protein expression. No changes in MCT8 expression (protein and mRNA). DEHP treatment at 400 μM significantly reduced the consumption of T3 and T4 in the culture medium
Ťupová et al. (2020)	Ritonavir and Elacridar	A431 cells	OATP1A2	≥ 3	5–20 min	Ritonavir (10 μM) and Elacridar (2 μM)	Ritonavir and elacridar are potent inhibitors of OATP1A2 activity
Tetro et al. (2021)	Antiepileptic Drugs	BeWo cells	LAT2	6	2 days and 5 days	1. Valproate: 42,83 and 166 µg/ml 2. Levetiracetem 10 and 30 µg/ml 3. Carbamazepine 6 and 12 µg/ml 4. lamotrigine 3 and 12 µg/ml 5. Lacosamide 5,10 and 20 µg/ml	1. Valproate highest dose (166 µg/ml) increase LAT2 mRNA 2. Levetiracetam (30 µg/ml) decreased LAT2 mRNA 3. Carbamazepine (12ug/ml) decreased LAT2 mRNA 4. Lamotrigine and Lacosamide had no effect
Li et al. (2022)	Cadmium	JEG-3 cells	TRA, TRβ and Dio 2	3	0 h, 6 h, 12 h and 24 h	20 μM at different time points	Following cadmium exposure: 1. mRNA levels of Dio2 and TRα were decreased 2. Protein expressions of Dio2 and TRα were downregulated 3. Nuclear translocation of TRα and TRβ were decreased
Young et al. (2022)	Nicotene	HTR8/SVneo (CRL-3271)	TTR	4 to 8	0-24 h	1 μM, 10 μM and 100 μM	Nicotine treatment significantly reduced the uptake of AlexaTTR-T4
Paul et al. (2023)	Cortisone	hTERT (Sw.71)	DIO2	3 to 4	6 and 24 h	Cortisone, 500 ng/mL	Cortisone had decreased in DIO2 mRNA expression, a result consistent with the in vivo findings

Abbreviations: *DEHP* Di(2-ethylhexyl) phthalate, *DIO3* Deiodinase 3, *DIO2* Deiodinase 2, *LAT1* L-Type Amino Acid Transporter 1, *MCT8* Monocarboxylate transporter 8, *PCB* Polychlorinated biphenyls, *rT3* Reverse Triiodothyronine, *SULT* Sulfotransferase, *BDE* Tetrabromodiphenyl ether, *TR* Thyroid hormone receptor, *T4* Thyroxine, *TBP* Tribromophenol, *T3* Triiodothyronine

**Table 5:** Receptor binding assays

References	Chemical	Assay	Thyroid hormone Carrier	Number of groups	Exposure dose	Main finding
Cotrina et al. (2022)	UV-filters and paraben preservatives	Qualitative assay of T4 binding by gel electrophoresis and competition binding studies	TTR	15	Started with 667 μM compound and then varied the concentration for disassociation curves etc.	UV-Filters (BP1, BP2, and 4HB) and the two parabens (MePB and BzPB) displaced T4 from TTR. BP2 and MePB only partially displaced T4 from TTR, whereas AvoBP, EHMC, and 5Me–1H-BT did not influence TTR/T4 binding. None of the compounds achieved higher TTR binding affinities than T4
Dong and Wade (2017)	Flame retardants, pesticides and plasticizers	New assay: MDCK cells overexpressing human MCT8 gene were used in a high throughput screening assay which detects the inhibitors of T3 uptake	MCT8	10	Final concentration of tested chemicals: 1000, 500, 250, 125, 62.5, 31.25, 15.63 and 7.82 mΜ	BPA inhibited T3 uptake. Co-treatment with BPA re-duced T3 uptakes to approximately 60% and 40% of the control at concentration of 125 μM and 250 μM, respectively
Zhao et al. (2017)	Organophosphorus flame retardants	8-anilino-1-naphtalenesulfonic acid ammonium salt − TTR (ANSA − TTR) fluorescence displacement assay	TTR	5	Not specified	The IC50 values (concentration required for 50% inhibition) were: EHDPP: 8340 ± 738 nM TPHP: 10,029 ± 1083 nM TBP: 31,159 ± 2129 nM These values were much higher than that of T4 (thyroxine), which had an IC50 of 291 ± 12 nM, showing that T4 bound more readily than these other compounds

Abbreviations: *MCT8* Monocarboxylate transporter 8, *TTR* Transthyretin

### Quality and risk of bias assessment

Separate quality/risk of bias assessment tools were used, depending on whether the study was epidemiological, in vivo, or in vitro. The quality assessment evaluation criteria were markedly more in-depth for the in vitro studies than for the in vivo and epidemiological studies (Supplemental Excel File). Quality assessment was performed on the whole published manuscript, regardless of whether only one study within the manuscript was relevant, by three reviewers.

For epidemiological studies, a quality/risk of bias assessment was performed using the National Institutes of Health (NIH) Quality Assessment Tool for “Observational Cohort and Cross-Sectional Studies” and “Case–Control Studies”. The evaluation was composed of 12–14 questions and was designed to assess bias in epidemiological studies by assessing the study population, inclusion and exclusion criteria, time from exposure to measurement, exposure assessment, blinding, and statistical analysis. Responses to questions were categorised as “Yes” (indicating low bias risk), “No” (suggesting high bias risk), or "Unclear" (denoting uncertain bias risk). A “No” response to any relevant question was interpreted as a high risk of bias for that entry.

The SYstematic Review Centre for Laboratory Animal Experimentation (SYRCLE) tool was used to assess the potential for bias in in vivo studies (Hooijmans et al. 2014). The evaluation consisted of ten questions which encompassed various types of bias, including selection, performance, detection, attrition, and reporting. Other sources of bias, such as contamination and the addition of new animals to groups, were also examined. The SYRCLE tool provides questions and judgement criteria to evaluate different types of bias. Responses to the questions were the same as those used in the NIH Quality Assessment Tool.

The Peer-Review In Vitro Appraisal Tool (PRIVAT) was used to assess the quality and risk of bias in in vitro studies (Whaley et al. 2024). The evaluation was composed of 26 questions and assessed the objectives and goals, experimental setup, power and replicates, bias, result generation, reporting, and interpretation of the results. The original scale used was for reviewers to assess the quality of manuscripts during the peer-review process and used scales, in response to questions such as “Critical issues that are grounds for rejection”, “Major issues that require reanalysis”, “Moderate issues that can be resolved via revision”, “Minor issues that can be resolved via revision”, “and No issues, no revisions necessary”. Here, they were replaced with the same used by the SYRCLE tool and NIH Quality Assessment Tool; namely “Yes” (indicating low bias risk), “No” (suggesting high bias risk), or “Unclear” (denoting uncertain bias risk).”

## Results

### Summary of the characteristics and quality of the included studies

Among the 1863 articles identified by PubMed, 24 satisfied the inclusion criteria (Fig. 1). The characteristics of the included studies are summarised in Tables 2, 3, 4 and 5. The studies were presented according to epidemiological (four studies), in vivo (seven studies), or in vitro (15 studies) methods; three of the 15 in vitro studies were binding assays. Two publications (Li et al. 2022; Paul et al. 2023) had relevant in vivo and in vitro components, and these experiments are presented separately in the respective tables and quality assessment strategies.

Briefly, there were four epidemiological studies, of which half were case–control studies (Li et al. 2022; McColl et al. 2023) and the other half were cohort studies (Leonetti et al. 2016; Kim et al. 2019). Three of the four studies observed differences in responses to chemicals due to infant sex, whereas one study did not investigate whether infant sex influenced the outcomes. Overall, the studies were of good quality, with responses as mainly “Yes” to the questions in the NIH Quality Assessment Tool. However, none of the studies justified the sample size chosen, and it was unclear if blinding occurred in any of the studies (Supplemental Excel File).

All seven in vivo studies were conducted in rodents. Four of the studies observed differences in responses to chemicals due to infant sex (Shukla et al. 2011; Wang et al. 2017; Guo et al. 2020; Paul et al. 2023) and three studies did not investigate this (Meerts et al. 2002; Yu et al. 2018; Blanco-Castañeda et al. 2022). In the quality assessment, there was uncertain risk bias in many publications with “unclear” being the predominant answer to most questions. Three publications did not adequately address the reason for incomplete or missing data (Wang et al. 2017; Yu et al. 2018; Paul et al. 2023), suggesting reporting bias (Supplemental Excel File).

There were 15 relevant in vitro studies, of which three were binding assays. All studies, except one (Gregoraszczuk et al. 2014), used placental tumour cell lines (JAR. BeWo, JEG-3, and A431) or continuous cell lines (HTR-8 and hTERT); Gregoraszczuk et al. 2014) used placental explants. In the quality assessment, nearly all studies scored “yes” for “Objectives and knowledge goals” and “Experimental set-up”. However, for “Power and replicates” and “Safeguards against systematic error”, “Unclear” was the predominant answer, suggesting an unknown bias. “Generation and reporting of results” had mixed results. However, none of the publications tested for normality before choosing a parametric/non-parametric test, and no study mentioned how outliers were excluded, if any. There were mixed qualities for “Interpretation of results into findings and conclusions” with the major problem being insufficient information on the study limits (Supplemental Excel File).

### Main findings

#### Persistent organic pollutants

POPs are chemical compounds that remain in the environment, accumulate in organisms, and threaten both human health and ecosystems (ECHA 2019). Chemicals, such as organochlorine pesticides, polybrominated diphenyl ether (PBDE) flame retardants, polychlorinated biphenyls (PCBs), perfluorooctanoic acid (PFOA), and perfluorooctane sulfonate (PFOS) are categorised as POPs. In Korea, an observational cohort study was conducted to investigate the relationship between maternal serum POP concentration and methylation of placental DIO3 and monocarboxylate transporter 8 (MCT8) (Kim et al. 2019).

DIO3 catalyses the inactivation of T4 and T3. Increased methylation of DIO3 could potentially reduce its expression, which may affect the conversion of T4 and T3 to inactive forms (Kim et al. 2019). MCT8 encodes a thyroid hormone transporter, and the methylation of MCT8 can affect its expression and potentially disrupt the passage of T4 from the mother to the foetus (Kim et al. 2019). In this study, a noticeable sex-specific pattern emerged in the epigenetic regulation of genes linked to the thyroid gland. Several maternal serum POPs [dichlorodiphenyldichloroethylene (DDE), dichlorodiphenyltrichloroethane (DDTs), Organochlorine Pesticides (OCPs), and BDE-47], were positively associated with DIO3 methylation in the placentas of female infants. In the placentas of male infants, only DDT and OCPs were positively associated with MCT8 methylation.

**Fig. 2:**
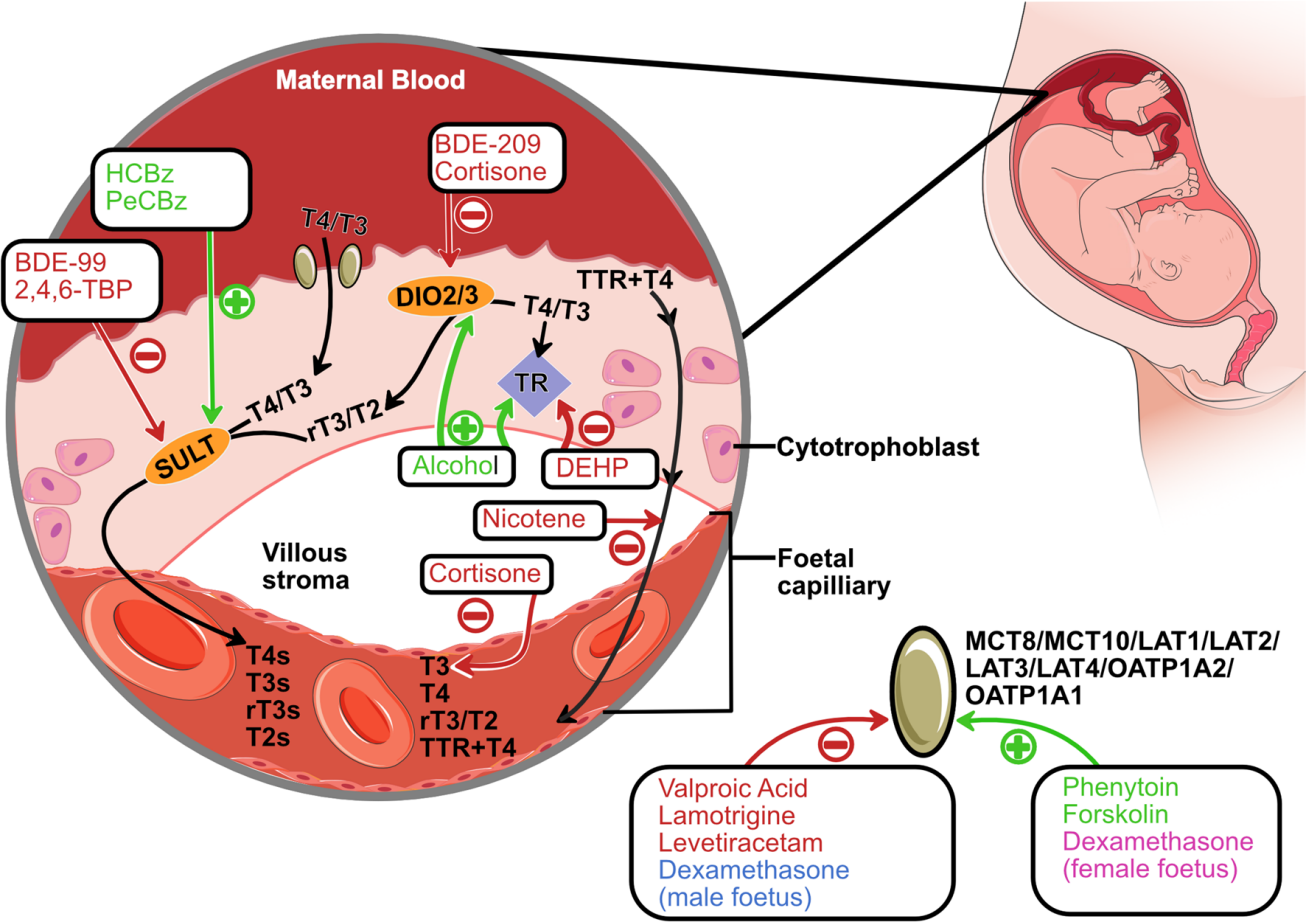
Adapted from Zuñiga et al. (2022), showing the proposed transfer of thyroid hormone across the placenta and how chemicals can interfere with this process. The chemicals listed showed changes at the protein level in the studies presented in the systematic literature review (foetal sex not mentioned unless the effect was opposite in males and females). Thyroid hormones cross the placenta through several transporters, including Monocarboxylate transporters (MCT8, MCT10), L-type amino acid transporters (LAT1, LAT2) and Organic anion transporting polypeptides (OATP1A2, and OATP4A1). Once inside the placenta, the hormones signal through the thyroid hormone receptors (TR), facilitating regulatory functions within the trophoblast. In addition, the placental enzymes deiodinases (DIO2 and DIO3) can convert thyroid hormones into more active forms (T4 to T3) or inactive forms (T4/T3 to rT3/T2). Trophoblast cells produce and release transthyretin (TTR), a carrier protein that binds to T4, forming a complex that is absorbed by trophoblast cells. Besides deiodination, which regulates hormonal activity, thyroid hormones can also undergo sulfation through sulfotransferases (SULT1A1 and SULT1A3). This process contributes to the presence of T4S, T3S, rT3S, and T2S in foetal circulation. The figure was partly generated using Servier Medical Art. Abbreviations: 2,4,6-Tribromophenol (2,4,6-TBP), 3,3',5-Triiodothyronine (T3), 3,3',5-Triiodothyronine sulfate (T3s), 3,3',5,5'-Tetraiodothyronine (T4), 3,3',5,5'-Tetraiodothyronine sulfate (T4s), 3,3'-Diiodothyronine sulfate (T2s), 3,5,3'-Triiodothyronine reverse (rT3s), 2,2',4,4',5-Pentabromodiphenyl ether (BDE-99), Deiodinase type 2 (DIO2), Deiodinase type 3 (DIO3) Di(2-ethylhexyl) phthalate (DEHP), Hexachlorobenzene (HCBz), L-type amino acid transporter 1(LAT1), Monocarboxylate transporter 8 (MCT8), Organic anion transporting polypeptide 1a1 (OATP1a1), Pentachlorobenzene (PeCBz) Sulfotransferase (SULT), Thyroid hormone receptor (TR), Transthyretin (TTR)

Gregoraszczuk et al. (2014), investigated the accumulation, metabolism, and effects of hexachlorobenzene (HCBz) and pentachlorobenzene (PeCBz) on the activity and expression of SULT1A in placental explants. HCBz increased SULT1A protein expression at all doses at 48 and 72 h following exposure to all tested does (0.02, 0.2, and 2 ng/ml) and stimulated SULT1A activity at 24 h for lower doses (0.02 and 0.2 ng/ml). Similarly, PeCBz increased SULT1A expression at 0.2 ng/ml at all time points (24, 28 and 72 h) and stimulated SULT1A activity (0.02 ng/ml and 0.2 ng/ml). These findings suggest that both HCBz and PeCBz can affect SULT1A activity and expression in placental tissue (Fig. 2); however, their impact on thyroid hormone homeostasis has not been addressed.

In rats, the effects of prenatal exposure to the PCB metabolite 4-OH-2,3,3',4',5-pentachlorobiphenyl (4-OH-CB107) on maternal and foetal thyroid hormone status and metabolism were investigated (Meerts et al. 2002). Pregnant rats were exposed to 4-OH-CB107 (5 mg/kg body weight per day) via oral dosing from gestational days 10–16. Placental thyroid hormone metabolism and transport markers were not examined in this study. Instead, foetal plasma was analysed, and on gestational day 20, following intrauterine exposure to the PCB metabolite, foetal total thyroxine (TT4) levels were decreased by 89% and foetal T4 levels were reduced by 41%. The foetal plasma TSH levels significantly increased by 124%. Sex differences were not explicitly studied or reported. However, the study concluded that prenatal exposure to 4-OH-CB107 leads to its accumulation in foetal tissues and significantly disrupts foetal thyroid hormone homeostasis, which could have implications for foetal development, particularly for neurodevelopment.

#### Flame retardants

Brominated flame retardants (BFRs) are a class of chemicals that are added to various consumer products to reduce their flammability. Some flame retardants are classified as POP’s, but not all are (Environment 2017), and for this reason, they are discussed separately from POP’s. Four studies investigated the effects of flame retardants on placental transporters of thyroid hormones.

In a cohort study in the USA, Leonetti et al. (2016) investigated the relationship between BFRs in the placenta and activities of DIO3, SULT, and TH (Leonetti et al. 2016). The majority of BFRs detected in the placenta were BDE-47, BDE-100, BDE-99, BDE154, BDE-153, BDE-209, and 2,4,6-tribromophenol (2,4,6-TBP). BFRs accumulated differently in placentas based on foetal sex, with higher concentrations in male placentas than in female placentas. For instance, male placental 2,4,6-TBP concentration was nearly double that of females. There was no relationship between maternal serum BFR concentrations and infant sex. Sex differences were also observed in the responses of placental thyroid hormone carriers to BFRs. Among males, several PBDE congeners were positively correlated with T3 SULT activity, whereas BDE-99 was negatively associated with T3 SULT activity (Fig. 2) among females. These differences observed between sexes indicate that BFRs could potentially have different effects on thyroid hormone function in male and female foetuses and infants.

BFRs have also been shown to affect placental TH enzyme levels in vitro. SULT enzyme inhibition in response to BFRs (2,4,6-TBP, 3-OH-BDE-47, or 6-OH-BDE-47) has also been observed in vitro (Leonetti et al. 2018). 2,4,6-TBP was the most potent inhibitor of SULT activity (Fig. 2), reducing it by up to 86% after 24 h of exposure to 1.0 μM in BeWo cells. In JEG-3 cells, Qin et al. (2021) found that low doses of BDE-209 (0.3–1.0 μM) decreased the expression of DIO3 at both the gene and protein levels (Fig. 2) (Qin et al. 2021). This BDE-209-mediated downregulation of placental DIO3 expression in JEG-3 cells may be due to changes in miRNA expression (specifically miR668-3p and miR409-3p) and DNA methylation patterns in the Dlk1–DIO3 imprinted domain following BDE209 exposure. BDE209 exposure (0.3 μM) also led to a disturbance in extracellular thyroid hormone equilibrium, with significant increases in extracellular T4 (indicating lower DIO3 and DIO2 activity) and decreased rT3. BDE-209 (1uM) increased DIO2 mRNA expression, resulting in a significant increase in extracellular T4 levels (Qin et al. 2021). Qin et al. (2021) concluded that environmental exposure to BDE209 during pregnancy may cause thyroid hormone interference in the foetus by affecting placental DIO3 expression. In addition, miR409-3p and miR668-3p may serve as potential biomarkers for thyroid hormone disturbance and developmental toxicity induced by in utero exposure to PBDEs.

Finally, the binding affinity of the flame retardants to TTR, a thyroid hormone-binding protein that chaperones T4 and protects it from inactivation, was studied. Structurally similar endocrine-disrupting chemicals (EDCs) can bind TTR and potentially disrupt thyroid hormone homeostasis (Bagga et al. 2023). In an 8-anilino-1-naphtalenesulfonic acid ammonium salt − TTR (ANSA − TTR) fluorescence displacement assay, Zhao et al. (2017) studied the binding affinity of organophosphorus flame retardants [2-ethylhexyldiphenyl phosphate (EHDPP), Triphenyl phosphate (TPHP) and 2,4,6‐Tribromophenol (TBP)] to TTR. The binding affinity was much lower than that of T4, suggesting low binding competition for T4 (Zhao et al. 2017).

#### Endocrine disrupting chemicals

EDCs interfere with the endocrine system of the body, which leads to adverse health outcomes, reproductive impairment, cognitive deficits, and obesity (La Merrill et al. 2020). People are exposed to a variety of EDCs throughout their lives from a myriad of sources, including food and beverage packaging, personal care products, and other consumer products (La Merrill et al. 2020).

Di-(2-ethylhexyl) phthalate (DEHP) is an endocrine-disrupting chemical (ECHA 2023) and a plasticiser that is commonly found in plastic consumer products. The effects of DEHP on thyroid hormone homeostasis were studied, both in vivo and in vitro. Mice were exposed to DEHP via oral gavage throughout pregnancy to investigate the role of placental thyroid hormone receptor (TR) signalling in DEHP-induced (50 and 200 mg/kg/d) intrauterine growth restriction (IUGR) (Yu et al. 2018). Gestational DEHP exposure significantly reduced foetal weight in a dose-dependent manner, but sex differences were not studied. There were no effects on the foetal total triiodothyronine (TT3) or thyroxine (TT4) levels. However, placental mRNA expression of Tra1 and Trb1 decreased following DEHP exposure, and nuclear translocation of the placental nuclear receptors TRA1 and TRB1 was suppressed, suggesting decreased TR signalling. One could argue that daily oral gavage of pregnant mice may cause marked stress and other confounding physiological changes (Brown et al. 2000; Hoggatt et al. 2010). Furthermore, it was unclear whether the vehicle control group received oral gavage, as the study only mentioned that DEHP was administered by oral gavage to the treatment groups. As the hypothalamic–pituitary–adrenal and the hypothalamic–pituitary–thyroid axes are involved in stress responses (Anifantaki et al. 2021), it is possible that some of these findings may be due to the stress induced by oral gavage rather than DEHP alone. However, DEHP has been shown to potentially affect thyroid hormone transport through the placenta in vitro (Du et al. 2020).

The potential mechanisms of DEHP-induced disruption in placental thyroid hormone transport were studied in vitro using two human placental trophoblastic cell lines, HTR-8/SVneo cells and JEG-3 cells (Du et al. 2020). DEHP treatment decreased TTR protein levels in the medium and decreased TTR internalisation in both cell types (Fig. 2). In HTR-8/SVneo cells, DEHP downregulated TTR mRNA expression in a dose-dependent manner, with significant decreases observed at 100 and 400 μM. These findings suggest that DEHP affects TTR at multiple levels, including its internalisation, gene expression, and protein production, which could potentially affect thyroid hormone transport through the placenta.

Lastly, the effect of fenvalerate, a synthetic pyrethroid insecticide with endocrine-disrupting properties, on IUGR and placental thyroid hormone receptor signalling was studied in mice (Wang et al. 2017). Dams were exposed to fenvalerate throughout pregnancy via daily oral gavage, and the controls were treated with vehicle via daily gavage. Placental Tra1 and Trb1 mRNA expression was down-regulated in fenvalerate-exposed mice at all doses (0.2, 2.0, and 20 mg/kg) and the nuclear translocation of TRB1 was decreased in the highest dose group (20 mg/kg). Interestingly, maternal fenvalerate exposure induced foetal IUGR in a sex-dependent manner, with reduced weight in male foetuses exposed to all doses of fenvalerate throughout pregnancy, and IUGR was only observed in female foetuses from dams exposed to the highest dose (20.0 mg/kg). Overall, this study provides evidence that maternal fenvalerate exposure induces foetal IUGR by disrupting placental thyroid hormone receptor signalling.

#### Addictive substances

Addictive substances, such as tobacco, nicotine, and alcohol, can have significant effects on foetal development and placental function, including effects on thyroid hormone transport and metabolism across the placenta (Shukla et al. 2011). Using rats, Shukla et al. (2011) aimed to identify candidate placental biomarkers for intrauterine alcohol exposure in an animal model of foetal alcohol spectrum disorders (Shukla et al. 2011). In the placentae of alcohol-exposed foetuses, DIO3 and TRA1 were significantly increased at both the mRNA and protein levels (Fig. 2) and this was similar for both male and female foetuses. This could potentially lead to reduced thyroid hormone passage to the foetus and contribute to the physiological and cognitive deficits observed in foetal alcohol spectrum disorders (Shukla et al. 2011).

A case**–**control study in Alaska explored whether smoking cigarettes or tobacco chewing during pregnancy is associated with changes in the expression of solute carrier transporters in the placentas (McColl et al. 2023). Only oral tobacco use was associated with significantly increased levels of LAT1 mRNA expression in the placenta, with no effect on protein expression. When protein expression was analysed for male and female foetuses separately, LAT1 protein expression was significantly decreased in women (maternal blood) who used oral tobacco carrying female foetuses. It is unclear whether tobacco affects thyroid hormone signalling in the foetus. However, in vitro studies using the immortalised human placental trophoblast cell line HTR8 and nicotine (1, 10, and 100 μM) showed reduced uptake of the TTR–T4 complex by trophoblasts (Fig. 2). Molecular dynamic modelling suggests that nicotine binds to the TTR–T4 complex, increasing its stability and potentially reducing its ability to enter trophoblasts.

#### Pharmaceuticals

##### Antiepileptic drugs

Women with epilepsy may be required to take antiepileptic drugs (AEDs) during pregnancy. Although uncontrolled seizures are associated with significant risks to both maternal and foetal health (Chen et al. 2009; Kemp et al. 2025), AED exposure can adversely affect the foetus (Tomson and Battino 2012). Between 2006 and 2016, approximately 1.5% of women took AED during pregnancy (Cohen et al. 2020). Ongoing research is focused on understanding AEDs and identifying safer options for pregnant women with epilepsy. Three studies have investigated the effects of AEDs on thyroid hormone metabolism and transport across the placenta.

Rubinchik-Stern et al. (2015) studied how AEDs alter placental carrier expression in BeWo cells (Rubinchik‐Stern et al. 2015). Here, the protein expression levels of LAT1 and members of the organic anion transporting polypeptides (OATP1A2 and OATP4A1) were measured. Levetiracetam (30 µg/ml) decreased the expression levels of all three carriers (LAT1, OATP1A2, and OATP4A1) (Fig. 2). Phenytoin (10 and 20 µg/ml) decreased LAT1 protein expression, and OATP1A2 protein expression decreased after exposure to lamotrigine (3 µg/ml, but not at 12 µg/ml) but a dose–response was not observed. Valproic acid (42 µg/ml only) decreased OATP4A1 expression (Fig. 2), but there was no dose response. No images of western blots are shown in the results or supplemental section. As a result, although this study scored well in the PRIVAT quality assessment tool, with “Yes”, there is a question regarding the reliability of the protein expression results.

Several years later, the same research group published another study that extended their analysis to heterodimeric placental carriers in BeWo cells, including LAT2 (Tetro et al. 2021). However, the analysis only examined the mRNA expression levels. The highest dose of valproate (166 µg/ml) increased LAT2 gene expression, while carbamazepine (12 µg/ml) and (30ug/ml) levetiracetam decreased LAT2 gene expression; neither lamotrigine nor lacosamide had an effect. These studies (Rubinchik‐Stern et al. 2015; Tetro et al. 2021) provide some insight into how AEDs alter the expression of placental carriers involved in the transport of thyroid hormones which could affect foetal development and drug exposure.

Levetiracetam administration during pregnancy and its effect on placental transporters was further studied by Blanco-Castañeda et al. (2022) in a murine model (Blanco-Castañeda et al. 2022), but sex differences were not studied. Pregnant BALB/c mice were administered 100 mg/kg/day levetiracetam by oral gavage, which is the highest dose at which the least foetal absorption was not observed. It is unclear when the dosing started, but it ended at either D13 (middle gestation) or D18 (late gestation). On GD 18, the gene expression of both Lat1 and Oatp4a1 was significantly higher in the group treated with levetiracetam than in the control group. The foetuses in the levetiracetam-treated group had IUGR, as evidenced by the significantly reduced weight, height, and width at both D13 and D18. The study suggests that LEV treatment alters placental transporter expression, particularly at late gestation, which may contribute to the observed intrauterine growth restriction.

##### Corticosteroids

The effects of foetal exposure to corticosteroids have been studied in the context of therapeutic treatment with dexamethasone during pregnancy (Guo et al. 2020) and maternal stress (Paul et al. 2023). Corticosteroids can be administered to mothers during early pregnancy to address issues such as recurrent miscarriages and congenital adrenal hyperplasias. In mid-late pregnancy, corticosteroid treatment can reduce the risk of serious complications in preterm births such as impaired breathing due to lung immaturity (Kemp et al. 2016). However, prenatal dexamethasone exposure can increase the risk of IUGR (Magann et al. 2017).

To understand the mechanisms underlying corticosteroid-induced IUGR, the effects of prenatal dexamethasone treatment on placental oxygen and nutrient transport were investigated in rats (Guo et al. 2020). Pregnant Wistar rats were administered dexamethasone from gestational days 9 to 20 via daily subcutaneous injections. The study found sex-specific differences in the placental response to dexamethasone, with the mRNA and protein expression of LAT1 increased in the female placenta and decreased in the male placenta (Fig. 2). However, dexamethasone exposure reduced foetal weight in a dose-dependent manner, irrespective of the foetal sex. LAT1 mediates the transport of large neutral amino acids and iodothyronines, including tri-iodo-l-thyronine (T3), across the placenta (Zuñiga et al. 2022). This study did not investigate thyroid hormone signalling itself but rather the effect of dexamethasone treatment on nutrient transporters.

Paul et al. (2023) investigated the effect of external corticosteroid administration on foetal thyroid signalling (Paul et al. 2023). Pregnant mice were exposed to cortisone from gestational days 13 to 17 or 19 through drinking water (50 μg/mL). Again, sex-specific differences in response to foetal cortisone exposure were observed. Dio2 mRNA was significantly decreased (two-fold reduction) in the placenta of female foetuses, while there was only a non-significant decrease in the number of males. Foetal circulating and placental T3 levels were significantly reduced in female foetuses from cortisone-treated dams, but not in males. Female embryos from cortisone-treated dams were significantly smaller than those from controls, a difference that was not observed in males. Sw.71 cells were used to study the possible mechanisms underlying these findings. Treatment with 500 ng/mL cortisone for 6 or 24 h significantly decreased the DIO2 protein expression (Fig. 2). Further mechanistic studies suggested that the downregulation of DIO2 is mediated by the glucocorticoid receptor. This study showed that maternal exposure to elevated corticosterone during pregnancy in mice leads to sex-specific alterations in placental and foetal thyroid hormone availability, with female offspring showing more pronounced effects, including reduced Dio2 expression, decreased T3 levels in the placenta and foetus (Fig. 2), and smaller foetal size. These effects may be mediated through glucocorticoid receptor signalling (Paul et al. 2023).

#### Miscellaneous

Forskolin, extracted from the roots of the Indian plant *Coleus forskohlii*, has been a staple in traditional medicine for hundreds of years. It directly activates adenylate cyclase, which is responsible for transforming ATP into cAMP (Balthasar et al. 2017). In BeWo cells, LAT1 and LAT2 mRNA expression significantly increased after 48 h of forskolin treatment (Fig. 2), an effect confirmed by immunoblotting for LAT1 (Balthasar et al. 2017). In JAR cells, the effects of different compounds on the LAT system were studied in leucine uptake experiments, and forskolin had no effect on the cellular uptake of leucine after 16 h of exposure. It is likely that exposure time plays a role in the different outcomes observed in these two studies, and it is possible that a longer exposure time of 48 h or more may have provided different outcomes for leucine uptake experiments.

Cadmium, a ubiquitous heavy metal, is the only metal studied in the context of placental thyroid signalling (Li et al. 2022). A case–control study conducted in China examined the relationship between preeclampsia, cadmium exposure, and placental thyroid hormone signalling; sex differences were not studied. Cadmium exposure had a negative impact on thyroid hormone signalling in the placenta. In preeclampsia placentas, which had higher cadmium levels, the mRNA expression and nuclear translocation of TRA were decreased. In addition, the protein expression of DIO2 was decreased in the placentas with higher cadmium levels. In JEG3 cells treated with cadmium chloride, the mRNA and protein levels of TRA and DIO2 were significantly decreased, and nuclear translocation of both TRA and TRB was decreased. Interestingly, cadmium exposure did not affect the expression of TRB in JEG3 cells, although its nuclear translocation was reduced. These findings suggest that cadmium exposure disrupts thyroid hormone signalling in placental cells by reducing the expression of key components (TRA and Dio2) and interfering with the nuclear translocation of thyroid hormone receptors. This disruption in thyroid hormone signalling may contribute to impaired placental angiogenesis observed in preeclampsia.

## Discussion

In this systematic review, a wide variety of chemicals have been identified to potentially disrupt placental thyroid hormone signalling, including POPs, flame retardants, EDCs, addictive substances, and pharmaceuticals, such as AEDs and corticosteroids. Most strikingly, the effects of some of these chemicals on the placenta are influenced by the sex of the foetus.

There is growing evidence that foetal sex influences placental responses to external stimuli in utero (Gabory et al. 2013; Rosenfeld 2015). Sex-specific epigenetic markers, along with a large number of X-linked genes that play a role in placental development, contribute to these differences (Gabory et al. 2013). These factors can affect the transcriptome and metabolome, ultimately influencing the placental response to xenobiotics (Cvitic et al. 2013; Buckberry et al. 2014; Saoi et al. 2020). When investigating the effects of chemicals on TH carriers and enzymes in the placenta, it was found that foetal sex influenced the degree of response (Shukla et al. 2011; Leonetti et al. 2016; Wang et al. 2017; Kim et al. 2019; Paul et al. 2023), the direction of the response (upregulation or downregulation) (Guo et al. 2020) or had no effect (Shukla et al. 2011). Different responses based on the foetal sex have been observed for specific chemicals. For example, arsenic exposure was found to strongly associate with AQP9 gene expression in female but not male placentas, suggesting sex-specific arsenic transport and developmental gene expression changes (Winterbottom et al. 2017). DNA methylation of AluYb8 is higher in males when exposed to xenoestrogen, whereas females exhibit no change (Vilahur et al. 2014). However, the interaction between environmental chemicals and sex in determining placental outcomes remains a critical gap in our understanding of developmental toxicology (Rosenfeld 2015).

These findings highlight the importance of considering foetal sex when studying chemical exposure and placental responses during pregnancy. Sex-specific placental adaptations likely contribute to differences in foetal programming and health outcomes between males and females. This review highlights the importance of studies investigating sex-specific accumulation and the effects of xenobiotics on the placenta and its subsequent health effects.

### Limitations

At present, in vitro research frequently relies on commercially sourced cell lines derived from placentas of unspecified sex, thus overlooking sex-specific effects. Most studies investigating the effects of chemicals on placental TH carriers and enzymes identified in this review were conducted in vitro (15 of the 24 studies analysed). Of these in vitro studies, the majority were conducted using choriocarcinoma cell lines (BeWo, JAR, and JEG-3). In addition to the lack of sex-specific data provided by these models, it is also necessary to consider differences in the proteome and transcriptome compared to the placenta. To determine how accurately these cell types reflect responses seen in primary placental tissue, Kruger et al. (2023) compared the global proteomes of placental cell lines to human placental tissue (a pool of 98 individual donors), focusing on xenobiotic metabolism pathways (Kruger et al. 2023). Overall, the levels of xenobiotic-metabolising proteins were lower in the cell lines than in the placenta, with JEG-3 cells being the most representative of the placenta. Laphen et al. (2025) investigated global transcriptomic differences across in vitro models and placental tissues (Lapehn et al. 2025). Using their “Comparative Transcriptomic Placental Model Atlas (CTPMA)” tool, we also examined the gene expression of TH transport carriers and metabolising enzymes (Supplemental Fig. 2). There were differences (log fold change > 2 or < − 2) in the gene expression of several TH carriers and enzymes between the placenta and the different cell lines, with BeWo cells having the least number of differentially expressed TH carriers and enzymes compared to the placenta.

These gene and protein expression differences between placental cell lines and the human placenta raise concerns regarding extrapolation of these results to humans. However, they still provide valuable information on how chemicals can potentially interfere with the transfer of TH across placentas. Despite the valuable insights gained from the reviewed studies, many relied on animal models or in vitro methods that may not fully replicate human placental processes. Moreover, the animal studies only included rodent species. Therefore, further research using human samples and considering real-world exposure scenarios is crucial. This systematic literature review also highlights the need for further studies using primary tissues, observing sex as a modifying factor, or using more advanced models to ensure the validity of these observations.

Publication bias may have influenced the findings, as studies with positive results are more likely to be published than those with null findings. Consequently, there may have been studies conducted that showed no effect of a chemical on placental thyroxine transporters and enzymes, but these were not published. Finally, the quality assessment revealed some methodological limitations in the included studies, such as lack of blinding and inadequate reporting of methods in some cases. These factors were taken into consideration when writing the manuscript.

## Conclusion

This review highlights that a wide range of environmental chemicals and medications can potentially disrupt the critical transfer of thyroid hormones from mother to foetus. Sex-specific effects have been observed in multiple studies, with male and female foetuses, or their placentas, showing different responses to chemical exposure. In some cases, the sex differences were limited to the degree of change, while in other cases, the same chemical yielded opposite effects depending on the foetal sex.

The clinical implications and long-term developmental effects of placental thyroid disruptions due to chemical exposure remain unclear. However, this is a significant concern, as thyroid hormones play a vital role in foetal development, particularly in neurodevelopment, growth, and metabolic regulation(O’Shaughnessy and Gilbert 2020). Further research is needed to fully elucidate the impacts on foetal thyroid status and development, considering foetal sex as a modifying factor, and using more advanced cellular models.

## Supplementary Information

Below is the link to the electronic supplementary material.

Supplementary file1 (XLSX 20 KB)

Supplementary file2 (DOCX 689 KB)

## Data Availability

All materials and data are provided in the manuscript and supplement. Additional informationis available from the corresponding author upon reasonable request.

## Abbreviations

BDE-99: 2,2',4,4',5-Pentabromodiphenyl ether
DIO2: Deiodinase type 2
DEHP: Di(2-ethylhexyl) phthalate
HCBz: Hexachlorobenzene
IUGR: Intrauterine growth restriction
LAT1: L-type amino acid transporter 1
MCT8: Monocarboxylate transporter 8
OATP1a1: Organic anion transporting polypeptide 1a1
PeCBz: Pentachlorobenzene
T3: 3,3',5-Triiodothyronine
SULT: Sulfotransferase
T4: Thyroxine
2,4,6-TBP: 2,4,6-Tribromophenol
TH: Thyroid hormone
TR: Thyroid hormone receptor
TRA: Thyroid hormone receptor alpha
TRB: Thyroid hormone receptor beta
TSH: Thyroid-stimulating hormone
TBG: Thyroxine-binding globulin
TT4: Total thyroxine
TTR: Transthyretin
T3: Triiodothyronine
