# Supplementary material for: Chemical disruption of placental thyroid hormone signalling: a systematic review that highlights sex-specific effects
Source: Arch Toxicol. 2025 Sep 25;100(1):75–93. doi: 10.1007/s00204-025-04203-z (PMC12858585; doi:10.1007/s00204-025-04203-z)
Supplement: Supplementary file 2 — Supplementary file2 (DOCX 689 KB) [file 204_2025_4203_MOESM2_ESM.docx]

# **Chemical Disruption of Placental Thyroid Hormone Signalling: A Systematic Review that Highlights Sex-Specific Effects**

J. Swan^1^, D. Zhurenko^1^, K.M. Huttunen^1^, J. Rysä^1^

**Supplemental data**

Supplemental Table 1 Methods: Search Terms for “The effect of any chemical exposure on thyroid hormone regulators within the fetoplacental unit”, Search date: February 19, 2024

|  |  |
| --- | --- |
|  | **PubMed/Medline** |
| #1 | fetoplacenta*[tw] OR foetoplacenta*[tw] OR placenta*[tw] OR fetus*[tw] OR fetal*[tw] OR foetus*[tw] OR foetal*[tw] OR embryo*[tw] OR uterus*[tw] OR utero*[tw] OR pregnan*[tw] OR mother*[tw] OR maternal*[tw] OR prenatal*[tw] OR "pre natal*"[tw] |
| #2 | "umbilical cord*"[tw] OR "umbilical arter*"[tw] OR "umbilical vein*"[tw] OR "umbilical blood"[tw] OR "cord blood"[tw] |
| #3 | "embryonic membrane*"[tw] OR "extraembryonic membrane*"[tw] OR allantois[tw] OR amnion[tw] OR "amniotic membrane*"[tw] OR "amniotic fluid*"[tw] OR "chorioallantoic membrane*"[tw] OR chorion[tw] OR "chorionic villi"[tw] |
| #4 | #1 OR #2 OR #3 |
| #5 | "thyroid regulating"[tiab:~5] OR "thyroid regulation"[tiab:~5] OR "thyroid regulator"[tiab:~5] OR "thyroid regulators"[tiab:~5] OR "thyroid hormone regulat*"[tw] OR "thyroid regulat*"[tw] OR "thyroxine binding globulin*"[tw] OR "thyroxin binding globulin*"[tw] OR TBG[tw] OR transthyretin*[tw] OR TTR[tw] OR "monocarboxylate transporter 8"[tw] OR MCT8[tw] OR "MCT 8"[tw] OR "monocarboxylate transporter 10"[tw] OR MCT10[tw] OR "MCT 10"[tw] OR "aromatic amino acid transporter*"[tw] OR "system L"[tw] OR LAT1[tw] OR LAT2[tw] OR LAT3[tw] OR LAT4[tw] OR "LAT 1"[tw] OR "LAT 2"[tw] OR "LAT 3"[tw] OR "LAT 4"[tw] OR (transport*[tw] AND (monoiodotyrosine[tw] OR diiodotyrosine[tw] OR diiodothyronine[tw] OR T2[tw] OR "T 2"[tw])) OR "organic anion transporting polypeptide*"[tw] OR OATP1A2[tw] OR OATP1A4[tw] OR "OATP E"[tw] OR "OATP 1A2"[tw] OR "OATP 1A4"[tw] OR "deiodinase type 2"[tw] OR "deiodinase type 3"[tw] OR "deiodinase 2"[tw] OR "deiodinase 3"[tw] OR DIO2[tw] OR DIO3[tw] OR "DIO 2"[tw] OR "DIO 3"[tw] OR "cytosolic sulfotransferase*"[tw] OR SULT1A1[tw] OR SULT1A3[tw] OR "SULT 1A1"[tw] OR "SULT 1A3"[tw] OR "thyroid hormone receptor*"[ti] OR TRα1[tw] OR TRalpha1[tw] OR TRα2[tw] OR TRalpha2[tw] OR TRβ1[tw] OR TRbeta1[tw] |
| #6 | expos*[tw] OR predispos*[tw] OR "pre dispos*"[tw] OR chemical*[tw] OR substance*[tw] OR compound*[tw] OR pollut*[tw] OR toxic*[tw] OR toxin*[tw] OR hazard*[tw] |
| #7 | #4 AND #5 AND #6 |
| #8 | review*[tw] OR "meta analysis"[tw] OR editorial*[tw] |
| #9 | #7 NOT #8 |
| #10 | #9 AND english[la] |
|  | 701 results |
|  |  |
|  | **Scopus** |
| #1 | #1 TITLE-ABS-KEY(fetoplacenta* OR foetoplacenta* OR placenta* OR fetus* OR fetal* OR foetus* OR foetal* OR embryo* OR uterus* OR utero* OR pregnan* OR mother* OR maternal* OR prenatal* OR "pre natal*") |
| #2 | #2 TITLE-ABS-KEY("umbilical cord*" OR "umbilical arter*" OR "umbilical vein*" OR "umbilical blood" OR "cord blood") |
| #3 | #3 TITLE-ABS-KEY("embryonic membrane*" OR "extraembryonic membrane*" OR allantois OR amnion OR "amniotic membrane*" OR "amniotic fluid*" OR "chorioallantoic membrane*" OR chorion OR "chorionic villi") |
| #4 | #4 #1 OR #2 OR #3 |
| #5 | #5 TITLE-ABS-KEY((thyroid W/3 regulat*) OR "thyroxine binding globulin*" OR "thyroxin binding globulin*" OR TBG OR transthyretin* OR TTR OR "monocarboxylate transporter 8" OR MCT8 OR "MCT 8" OR "monocarboxylate transporter 10" OR MCT10 OR "MCT 10" OR "aromatic amino acid transporter*" OR "system L" OR LAT1 OR LAT2 OR LAT3 OR LAT4 OR "LAT 1" OR "LAT 2" OR "LAT 3" OR "LAT 4" OR (transport* AND (monoiodotyrosine OR diiodotyrosine OR diiodothyronine OR T2 OR "T 2")) OR "organic anion transporting polypeptide*" OR OATP1A2 OR OATP1A4 OR "OATP E" OR "OATP 1A2" OR "OATP 1A4" OR "deiodinase type 2" OR "deiodinase type 3" OR "deiodinase 2" OR "deiodinase 3" OR DIO2 OR DIO3 OR "DIO 2" OR "DIO 3" OR "cytosolic sulfotransferase*" OR SULT1A1 OR SULT1A3 OR "SULT 1A1" OR "SULT 1A3" OR "thyroid hormone receptor*" OR TRα1 OR TRalpha1 OR TRα2 OR TRalpha2 OR TRβ1 OR TRbeta1) |
| #6 | #6 TITLE-ABS-KEY(expos* OR predispos* OR "pre dispos*" OR chemical* OR substance* OR compound* OR pollut* OR toxic* OR toxin* OR hazard*) |
| #7 | #7 #4 AND #5 AND #6 |
| #8 | #8 TITLE-ABS-KEY(review* OR "meta analysis" OR editorial*) |
| #9 | #9 #7 AND NOT #8 |
|  | Limit to: English language |
|  | 408 results |
|  |  |
|  | **Web of Science** |
| #1 | TS=(fetoplacenta* OR foetoplacenta* OR placenta* OR fetus* OR fetal* OR foetus* OR foetal* OR embryo* OR uterus* OR utero* OR pregnan* OR mother* OR maternal* OR prenatal* OR "pre natal*") |
| #2 | TS=("umbilical cord*" OR "umbilical arter*" OR "umbilical vein*" OR "umbilical blood" OR "cord blood") |
| #3 | TS=("embryonic membrane*" OR "extraembryonic membrane*" OR allantois OR amnion OR "amniotic membrane*" OR "amniotic fluid*" OR "chorioallantoic membrane*" OR chorion OR "chorionic villi") |
| #4 | #1 OR #2 OR #3 |
| #5 | TS=((thyroid NEAR/3 regulat*) OR "thyroxine binding globulin*" OR "thyroxin binding globulin*" OR TBG OR transthyretin* OR TTR OR "monocarboxylate transporter 8" OR MCT8 OR "MCT 8" OR "monocarboxylate transporter 10" OR MCT10 OR "MCT 10" OR "aromatic amino acid transporter*" OR "system L" OR LAT1 OR LAT2 OR LAT3 OR LAT4 OR "LAT 1" OR "LAT 2" OR "LAT 3" OR "LAT 4" OR (transport* AND (monoiodotyrosine OR diiodotyrosine OR diiodothyronine OR T2 OR "T 2")) OR "organic anion transporting polypeptide*" OR OATP1A2 OR OATP1A4 OR "OATP E" OR "OATP 1A2" OR "OATP 1A4" OR "deiodinase type 2" OR "deiodinase type 3" OR "deiodinase 2" OR "deiodinase 3" OR DIO2 OR DIO3 OR "DIO 2" OR "DIO 3" OR "cytosolic sulfotransferase*" OR SULT1A1 OR SULT1A3 OR "SULT 1A1" OR "SULT 1A3") OR TI=("thyroid hormone receptor*") OR TS=(TRα1 OR TRalpha1 OR TRα2 OR TRalpha2 OR TRβ1 OR TRbeta1) |
| #6 | TS=(expos* OR predispos* OR "pre dispos*" OR chemical* OR substance* OR compound* OR pollut* OR toxic* OR toxin* OR hazard*) |
| #7 | #4 AND #5 AND #6 |
| #8 | TS=(review* OR "meta analysis" OR editorial*) |
| #9 | #7 NOT #8 |
|  | Limit to: English language |
|  | 754 results |

Supplemental Table 2 Summary of how chemicals affected placental membrane transporters and enzymes

Black: epidemiological study, Blue: in vivo studies and Green: in vitro studies

| ***Group*** | ***Chemical*** | ***membrane transporters*** | | | | | ***Enzymes*** | | | ***Carrier*** | | ***Receptor*** | |  |
| --- | --- | --- | --- | --- | --- | --- | --- | --- | --- | --- | --- | --- | --- | --- |
|  |  | **LAT2** | **LAT1** | **OATP1A2** | **OATP4A1** | **MCT 8** | **DIO2** | **DIO3** | **SULT** | **TTR** | **TTR-T4 complex** | **TRA** | **TRB** | |
| ***POPs*** | **HCBz** |  |  |  |  |  |  |  | **Protein  Activity** |  |  |  |  | |
|  | **PeCBz** |  |  |  |  |  |  |  | **Protein  Activity** |  |  |  |  | |
|  | **POPs** |  |  |  |  |  |  | ** Methylation (females)** |  |  |  |  |  | |
|  | **DDT and OCPs** |  |  |  |  | ** Methylation (males)** |  |  |  |  |  |  |  | |
| ***Flame retardants*** | **BDE** |  |  |  |  |  |  |  | **Activity  in males** |  |  |  |  | |
|  | **BDE-99** |  |  |  |  |  |  |  | **Activity  in females** |  |  |  |  | |
|  | **2,4,6-TBP** |  |  |  |  |  |  |  | **Activity ** | **Bind - lower affinity than T4** |  |  |  | |
|  | **BDE209** |  |  |  |  |  | **mRNA** | **Protein** |  |  |  |  |  | |
| ***EDC*** | **DEHP** |  |  |  |  |  |  |  |  | ** Protein** |  | ** mRNA  nuclear translocation** | ** mRNA  nuclear translocation** | |
|  | **fenvalerate** |  |  |  |  |  |  |  |  |  |  | ** mRNA** | ** mRNA** | |
| ***Chemicals of Dependance*** | **Alcahol** |  |  |  |  |  |  | **Protein** |  |  |  | **Protein** |  | |
|  | **nicotene** |  |  |  |  |  |  |  |  |  | ** stability of this complex, inhibiting its uptake into trophoblasts** |  |  | |
| ***Anti-Epeleptic Drugs*** | **VALPROIC ACID** | **mRNA** | **mRNA** |  | **Protein** |  |  |  |  |  |  |  |  | |
|  | **phenytoin** |  | **Protein** |  | **mRNA** |  |  |  |  |  |  |  |  | |
|  | **carbamazepine** | ** mRNA** |  | ** mRNA** | ** mRNA** |  |  |  |  |  |  |  |  | |
|  | **lamotrigine** |  | **mRNA** | **Protein** | ** mRNA** |  |  |  |  |  |  |  |  | |
|  | **Levetiracetam** | ** mRNA** | **Protein / mRNA** | **Protein** | **Protein / mRNA** |  |  |  |  |  |  |  |  | |
| ***Corticosteroids*** | **dexamethazone** |  | ** in females and  in males protein** |  |  |  |  |  |  |  |  |  |  | |
|  | **cortisone** |  |  |  |  |  | **mRNA** |  |  |  |  |  |  | |
| ***Miscellaneous*** | **forskolin** | **mRNA** | **mRNA and protein** |  |  |  |  |  |  |  |  |  |  | |
|  | **Cadmium** |  |  |  |  |  | **Protein Protein** |  |  |  |  | ** mRNA  nuclear translocation/ Protein** |  | |


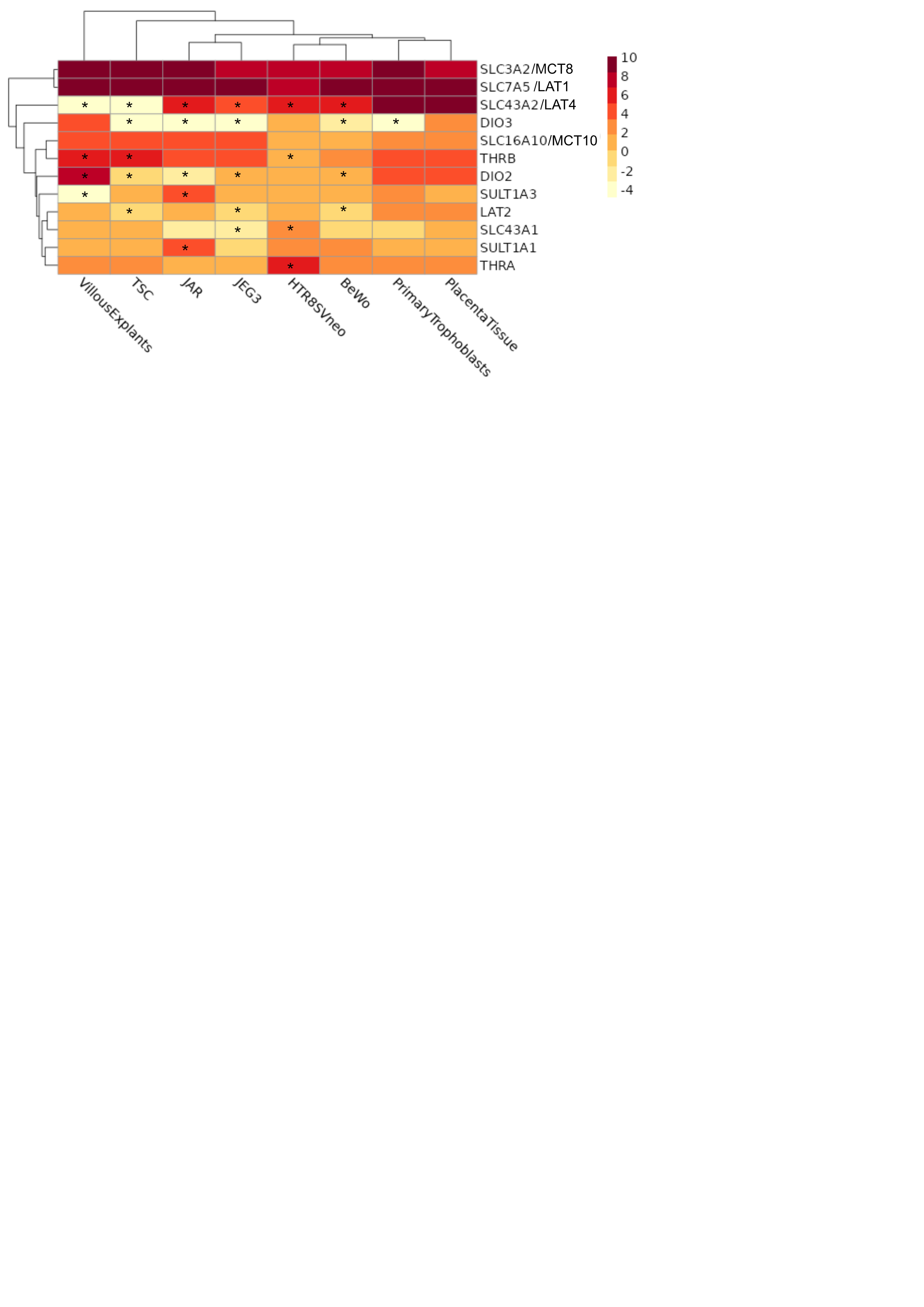


Supplemental Figure 1. The values in the heat map are batch corrected and normalized log counts per million (CPM). * Indicates log fold change for CANDLE/GAPPS placental samples to be greater than 2 or less than -2 (Lapehn et al. 2025). TSC are trophoblast stem cells.
